# Theta-Burst Stimulation-Induced Plasticity over Primary Somatosensory Cortex Changes Somatosensory Temporal Discrimination in Healthy Humans

**DOI:** 10.1371/journal.pone.0032979

**Published:** 2012-03-07

**Authors:** Antonella Conte, Lorenzo Rocchi, Andrea Nardella, Sabrina Dispenza, Alessandra Scontrini, Nashaba Khan, Alfredo Berardelli

**Affiliations:** 1 IRCCS Neuromed Institute, Pozzilli (IS), Italy; 2 Department of Neurology and Psychiatry, “Sapienza”, University of Rome, Rome, Italy; Katholieke Universiteit Leuven, Belgium

## Abstract

**Background:**

The somatosensory temporal discrimination threshold (STDT) measures the ability to perceive two stimuli as being sequential. Precisely how the single cerebral structures contribute in controlling the STDT is partially known and no information is available about whether STDT can be modulated by plasticity-inducing protocols.

**Methodology/Principal Findings:**

To investigate how the cortical and cerebellar areas contribute to the STDT we used transcranial magnetic stimulation and a neuronavigation system. We enrolled 18 healthy volunteers and 10 of these completed all the experimental sessions, including the control experiments. STDT was measured on the left hand before and after applying continuous theta-burst stimulation (cTBS) on the right primary somatosensory area (S1), pre-supplementary motor area (pre-SMA), right dorsolateral prefrontal cortex (DLPFC) and left cerebellar hemisphere. We then investigated whether intermittent theta-burst stimulation (iTBS) on the right S1 improved the STDT. After right S1 cTBS, STDT values increased whereas after iTBS to the same cortical site they decreased. cTBS over the DLPFC and left lateral cerebellum left the STDT statistically unchanged. cTBS over the pre-SMA also left the STDT statistically unchanged, but it increased the number of errors subjects made in distinguishing trials testing a single stimulus and those testing paired stimuli.

**Conclusions/Significance:**

Our findings obtained by applying TBS to the cortical areas involved in processing sensory discrimination show that the STDT is encoded in S1, possibly depends on intrinsic S1 neural circuit properties, and can be modulated by plasticity-inducing TBS protocols delivered over S1. Our findings, giving further insight into mechanisms involved in somatosensory temporal discrimination, help interpret STDT abnormalities in movement disorders including dystonia and Parkinson's disease.

## Introduction

Precise timing of sensory information is crucial for nearly every aspect of human perception and behavior. The physiological mechanisms underlying timing operations include afferent sensory input gating and a time-locked interplay between cortical and subcortical structures [1], [2]. An experimental approach for investigating how cerebral structures contribute to timing for sensory information entails studying the temporal threshold for perceiving two tactile stimuli applied to the skin as clearly distinct, namely the somatosensory temporal discrimination threshold (STDT). Despite inter-subject variability, most healthy individuals perceive two tactile stimuli as sequential when the interstimulus interval (ISI) exceeds 30–50 msec [3]. STDT testing activates neural processes involved in a sensory discrimination task uninfluenced by memory formation [3]–[5]. A functional magnetic resonance imaging (fMRI) study showed that STDT selectively activates the pre-supplementary motor area (pre-SMA) [6] supporting previous observations of altered STDT in patients with focal lesion of the SMA after neurosurgery [3]. Current knowledge therefore implies that the pre-SMA intervenes in the STDT, even though precisely how it contributes to STDT processing remains unclear.

Some evidence on the neural circuits involved in the STDT comes from previous studies showing that single-pulse transcranial magnetic stimulation (TMS) [7], [8] delivered to primary somatosensory cortex (S1) about 50 ms before the tactile stimuli impairs discrimination of two temporally separated stimuli. One way of investigating the role played by cortical areas on sensory processing is to deliver repetitive magnetic stimulation given as theta-burst stimulation (TBS) [9]. Unlike single-pulse TMS, TBS induces long-term changes in cortical responsiveness to external stimuli – namely, cortical synaptic plasticity. Although these effects vary among subjects they usually last less than 30 minutes [9]. Cortical responses to TBS differ according to the stimulus pattern: intermittent TBS (iTBS) elicits excitatory and continuous TBS (cTBS) inhibitory effects on cortical excitability. Whether plasticity inducing protocols such as TBS modulate the STDT remains unclear. Even though studies investigating temporal discrimination with a time estimation task suggest that the right dorsolateral prefrontal cortex (DLPFC) intervenes in cognitively controlled time measurements [10], [11], no evidence yet shows whether the cortical networks engaged by temporal discrimination tasks (time estimation tasks vs. STDT) overlap. Although STDT tasks, unlike time-estimation tasks [11], do not involve working memory, whether cTBS over the DLPFC leaves STDT values unchanged is unclear. Nor do we understand how cTBS over the pre-SMA might change expected STDT values. Previous temporal discrimination studies showed that the cerebellum intervenes in temporal processing at long ISIs (hundreds of milliseconds) [12] or during acquisition and coding of learned timing [13]. No study has yet shown whether cerebellar TBS intervenes in STDT entailing short ISIs thus clarifying whether temporal discrimination tasks using short and long ISIs activate different cortical and subcortical networks. Nor have previous studies compared how the various cortical (S1 but also non-primary sensory areas) and subcortical areas modulate the STDT within subjects. Answering these questions will help better understand the role played by the cerebral structures in the pathophysiology of the STDT alterations reported in dystonia and Parkinson's disease [5], [14]–[18].

In this study using a TBS protocol able to induce long-lasting changes in synaptic activity in the stimulated area in healthy subjects, we investigated whether the cortical (S1, pre-SMA, DLPFC) and subcortical (cerebellum) areas thought to intervene in other forms of temporal processing also play a role in controlling the STDT. To clarify whether cTBS over the pre-SMA changes the STDT directly by inhibiting the pre-SMA or indirectly by modulating the DLPFC via pre-SMA/DLPFC connections, we investigated the effects of cTBS over the DLPFC. To do so, using a within-subjects experimental design we applied cTBS to induce inhibitory effects on the right S1, pre-SMA, right DLPFC and left lateral cerebellum. STDT was tested on the cutaneous area of the index finger of the left hand. To ensure that we had correctly positioned the coil we used a neuronavigator system (S1, pre- SMA, DLPFC) and to check whether TBS effectively stimulated the S1 and cerebellum we also probed the somatosensory evoked potential (SEP) N20, N20-P25 and P25-N33 components after cTBS over the right S1 and assessed right primary motor area (M1) excitability after left cerebellar hemisphere stimulation. Because we found that cTBS over S1 altered the STDT whereas cTBS over the pre-SMA, DLPFC and left lateral cerebellum did not, and because in a previous study iTBS over S1 improved tactile spatial discrimination [19], we then investigated whether iTBS applied to the right S1 improved the STDT.

## Materials and Methods

A total 18 healthy volunteers, all right handed (to keep the sample as homogeneous as possible with respect to the hemispheric dominance), were enrolled after giving written informed consent. Of these 18 participants, 15 subjects underwent iTBS/cTBS over S1 and cTBS over left lateral cerebellum; 12 subjects underwent cTBS over the pre-SMA and DLPFC; 10 underwent control experiments (MEP and SEP); and 12 underwent TBS over all the cortical areas and cerebellum. Of the 18 subjects, 10 therefore completed all the experimental sessions including control experiments. The experimental procedures used here were carried out in accordance with the Declaration of Helsinki and approved by the institutional review board of the Department of Neurology and Psychiatry, “Sapienza” University of Rome.

### Stimuli and STDT procedure

STDT was investigated by delivering paired stimuli starting with an inter-stimulus interval (ISI) of 0 msec (simultaneous pair), and progressively increasing the ISIs (in 10 msec steps) according to the experimental procedures already used in previous studies [14], [16], [18], [20]. Paired tactile stimuli consisted of square-wave electrical pulses delivered with a constant current stimulator (Digitimer DS7AH) through surface skin electrodes with the anode located 0.5 cm distally from the cathode. The surface skin electrodes were applied, on the left hand (index finger). We studied the left hand because ample evidence suggests that timing processes depend on a right hemispheric cortical network [1], [2], [10], [11], [21]–[24]. The stimulation intensity was defined for each subject by delivering series of stimuli at increasing intensity from 2 mA in steps of 1 mA; the intensity used for STDT was the minimal intensity perceived by the subject in 10 of 10 consecutive stimuli. Before starting STDT testing subjects familiarized themselves with the task and achieved a stable performance. Subjects were asked to report whether they perceived a single stimulus or two temporally separated stimuli by saying “one” or “two” after each stimulation. The first of three consecutive ISIs at which participants recognized the stimuli as temporally separated was considered the STDT. To keep subjects attention level constant during the test and to minimize the risk of perseverative responses, the STDT testing procedure included “catch” trials consisting of a single stimulus randomly delivered. Errors in which subjects reported two stimuli instead of one during the “catch” trials were recorded for each experimental session and entered in the data analysis. Each session comprised four separate blocks. The STDT was defined as the average of four STDT values, one for each block, and was entered in the data analysis.

### Transcranial magnetic stimulation

A Magstim Super Rapid magnetic stimulator (Magstim Company, Whitland, Wales, UK) connected to a figure-of-eight coil 90 mm in diameter was used to deliver rTMS over the right S1, pre-SMA, right DLPFC, left lateral cerebellum. For right S1 and left lateral cerebellum stimulation as well as checking coil positioning with a neuronavigator Polaris Vicra optical measurement system (Northern Digital Inc.) we checked stimulating protocol efficacy by measuring SEPs (S1) and MEPs (left lateral cerebellum).

rTMS was delivered using the “theta burst” stimulation (TBS) paradigm [9]. For cTBS to the right S1, pre-SMA, left lateral cerebellum and right DLPFC, three-pulse bursts at 50 Hz repeated every 200 ms for 40 s [9] were delivered at 80% active motor threshold (AMT) (600 pulses). For iTBS to the right S1, three-pulse bursts at 50 Hz were delivered in short trains lasting 2 seconds repeated every 10 seconds for 20 trains; iTBS also was delivered at 80% AMT (600 pulses). To determine the intensity of cTBS and iTBS, AMT was calculated during a 20–30% maximum voluntary contraction of the target muscle as the lowest intensity able to evoke a motor evoked potential (MEP) of at least 200 µV in five out ten consecutive trials. AMT was tested using a figure-of-eight coil placed over the first dorsal interosseus muscle (FDI) area in the right hemisphere for right S1, pre-SMA and right DLPFC stimulation, and over the motor cortex in the left hemisphere for left lateral cerebellar stimulation.

A monophasic Magstim stimulator connected to a figure-of-eight coil was used to deliver single transcranial magnetic stimulation (TMS) pulses over the FDI motor hot-spot on the right hemisphere to probe M1 excitability after left lateral cerebellum-cTBS.

### Cortical localization using the neuronavigation system

To ensure accurate coil positioning throughout the experiment we used a neuronavigator Polaris Vicra optical measurement system (Northern Digital Inc.) combined with the Softaxic Evolution navigator system (E.M.S., Bologna, Italy). Of the 18 subjects, 12 underwent an anatomical T1-weighted MRI scan. For the remaining 6 subjects we obtained an estimated individualized MRI scan in the Talairach Space. The software uses a set of digitized skull landmarks (nasion, inion, right and left preauricular points) and about 60 scalp points to provide a uniform representation of the scalp, which is then adapted to a normalized reference volume of highly detailed T1-weighted MRIs to obtain an estimated individualized MRI scan in the Talairach Space [25]. Previous studies demonstrated that the mean accuracy of the estimated MRI scans is comparable to the spatial resolution of TMS [26], [27].

### Main experiments

**cTBS/iTBS over right S1:** iTBS/cTBS was applied in 15 subjects over the right S1 cortex with the coil located according to Talairach coordinate reported in a previous study (x, y, z) = (48, −28, 54) [28]. The coil was held with the handle pointing back and 45° away from the midline.

**cTBS over pre-SMA:** For right pre-SMA stimulation, in 12 subjects, we used the Talairach coordinates (x, y, z) = (−4, 32, 51) previously indicated as corresponding to the pre-SMA [29], [30].

**cTBS over right DLPFC:** DLPFC stimulation was delivered in 12 subjects with the coil held with the handle pointing back and 45° away from the midline, and directed at the junction of the middle and anterior one-third of the middle frontal gyrus (Talairach coordinates (x, y, z) = (50, 30, 36) corresponding with posterior region of BA 9, which overlaps with the superior section of BA 46. This site was chosen according to information from studies about working memory and the DLPFC [31]–[33].

**cTBS over left lateral cerebellum:** To stimulate the left lateral cerebellum, cTBS was delivered in 15 subjects with the coil placed 1 cm inferior and 3 cm to the left of the inion. The coil was positioned tangentially to the scalp, with the handle pointing superiorly. According to previous studies this scalp site corresponds to the posterior and superior lobules of the lateral cerebellum [34], [35] and the coil orientation used allowed us to modulate contralateral M1 excitability [36], [37].

### Control experiments

**Effects of S1 cTBS on upper-limb SEP:** The efficiency of cTBS in stimulating S1 was assessed in 10 subjects by recording SEPs after electrical stimulation applied to the left median nerve at the wrist at 3 Hz with a pulse width of 0.2 ms. The intensity of stimulation was fixed at motor threshold and was checked throughout the experiment by monitoring the evoked electromyographic (EMG) response in the abductor pollicis brevis (APB) muscle. SEPs were recorded from scalp Ag–AgCl surface electrodes 2 cm posterior from C4 (parietal component) referred to frontal region (Fz) according to the 10–20 electrode system for EEG placement. Recordings were band-passed from 3 Hz to 1 kHz using a Digitimer. All data were collected at a sampling rate of 5 kHz for a 200 ms recording epoch beginning 20 ms before each stimulus. A total of 500 responses were averaged in each session. The SEP assessment lasted about 3 minutes. SEP N20-P25 and P25-N33 component amplitudes were measured peak to peak. SEP N20 amplitudes were also measured baseline-to-peak and data entered in a further statistical analysis.

**Effects of left lateral cerebellar-cTBS on M1 excitability:** The effectiveness of cTBS in activating left lateral cerebellum was assessed in 10 subjects by measuring MEP size after cTBS. Control MEPs were evoked by single TMS pulses over the FDI motor hot-spot of the right hemisphere and delivered with the Monophasic stimulator. The intensity of single TMS pulses was set to obtain a mean MEP size of about 1 mV peak-to-peak at baseline. This intensity was maintained unchanged throughout the experiment controlling for changes in the STDT after cTBS over the left lateral cerebellum. Twenty MEPs were measured peak-to-peak and averaged before the pre-cTBS STDT and ten minutes after cTBS stimulation.

### Electromyographic recording

The EMG activity was recorded through a pair of Ag/AgCl electrodes placed over the left FDI muscle in a belly-tendon fashion. Raw signal, sampled at 5 kHz with a CED 1401 A/D laboratory interface (Cambridge Electronic Design, Cambridge, UK), was amplified and filtered (bandwidth 20 Hz–1 kHz) with a Digitimer D 360 (Digitimer Ltd., Welwyn Garden City, Hertfordshire, UK). Data were stored on a laboratory computer for on-line visual display and further off-line analysis (Signal software, Cambridge Electronic Design, Cambridge, UK). To ensure complete target muscle relaxation throughout the experimental sessions we continuously monitored the EMG activity with audio and high-gain visual feedback.

### Experimental sessions

The study comprised four experimental sessions that took place at least two weeks apart. During each experimental session the subjects underwent the STDT study before cTBS (T0), and 5 (T1) and 15 minutes (T2) after cTBS. Upper-limb SEPs were recorded before (T0) and 10 minutes (T1_SEP_) after cTBS over the right S1. Upper-limb MEPs were recorded before (T0) and 10 minutes (T1_MEP_) after cTBS over the left lateral cerebellum. In a further experimental session, changes in STDT values were investigated before and after iTBS over the right S1.

### Statistical analysis

STDT values were tested with a separate repeated measures ANOVA with factor “time” (before and after cTBS: T0, T1, T2) as main factor for data collected in each experimental session (entering STDT values from 15 subjects for iTBS/cTBS over S1 and cTBS over the left lateral cerebellum and STDT values from 12 subjects for cTBS over the pre-SMA and DLPFC). To investigate within-subjects changes in the STDT values across the different cortical areas in the 12 subjects who underwent all the experimental sessions (all of them with their individualized MRI scan) we ran a further repeated measures ANOVA with factor “cortical areas” and “time” as main factors of analysis. To identify possible changes in STDT values at T0 in each subject across the five experimental sessions we ran a further repeated measures ANOVA. Greenhouse-Geisser's correction for non sphericity was applied when needed. To control for the effects of cTBS over the right S1, left lateral cerebellum, N20, N20-P25 and P25-N33 SEP amplitudes (for the S1 c-TBS experiment), and the 1 mV MEP amplitude (for left lateral cerebellar cTBS) were also tested with a repeated measure ANOVA with factor “time” (before and after cTBS: T0, T1) as main factor. Tukey's Honest significance difference was used for post hoc analysis. Because the number of errors during the “catch” trials are not continuous values, we ran a Friedman's repeated measures ANOVA and Wilcoxon's test for post hoc analysis. P values<0.05 were considered significant. All values are expressed as mean ± SE.

## Results

### Main experiments

**Figure 1:**
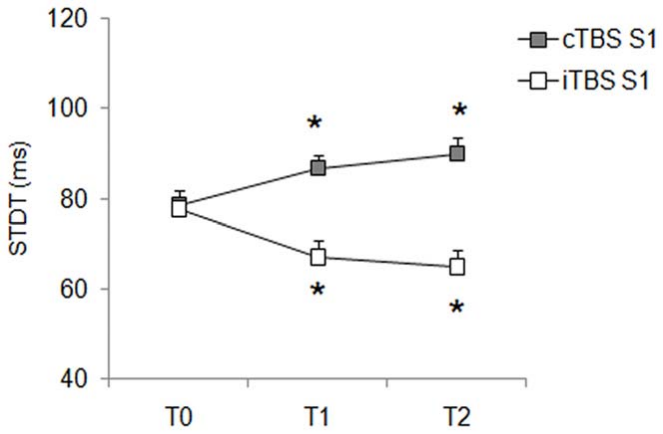
Changes in somatosensory temporal discrimination thresholds (STDT) induced by continuous theta-burst stimulation (cTBS) and intermittent theta-burst stimulation (iTBS) over primary somatosensory cortex (S1) in healthy subjects. Each point represents the mean; bars represent standard error. X axis: time: T0 (before cTBS/iTBS), T1 (5 minutes after cTBS/iTBS) and T2 (15 minutes after cTBS/iTBS). Y axis: STDT expressed in millisecond**s**. Asterisks indicate statistical significance.

**Effects of cTBS over the right S1 on the STDT:** Repeated measures ANOVA for STDT values after cTBS over S1 showed a significant effect of factor “time ” (F_(2,28)_ = 7.04; *P* = 0.003). Post hoc analysis showed that STDT significantly increased after cTBS (STDT at T0:75.8±3 ms vs. T1:86.7±4 and T2:87.8±4 ms) and the increase was significant at T1 (*P* = 0.03) and T2 (*P* = 0.004) (Figure 1). Friedman's repeated-measures ANOVA for number of errors during the STDT task showed no significant differences in the number of errors before and after cTBS.

**Effects of iTBS over the right S1 on the STDT:** Repeated measures ANOVA for STDT measured after iTBS over S1 showed a significant effect of factor “time ” (F_(2,28)_ = 12.74; *P* = 0.0001). Post hoc analysis showed that the STDT decreased significantly after iTBS and the decrease was significant at T1 (STDT at T0 = 77.7±4 ms vs. STDT at T1 = 67.1±3 ms; *P* = 0.0001) and T2 (STDT at T2 = 65±3 ms; *P* = 0.002) (Figure 1). Friedman's repeated-measures ANOVA for number of errors during the STDT task showed no significant differences in the number of errors before and after iTBS.

**Figure 2:**
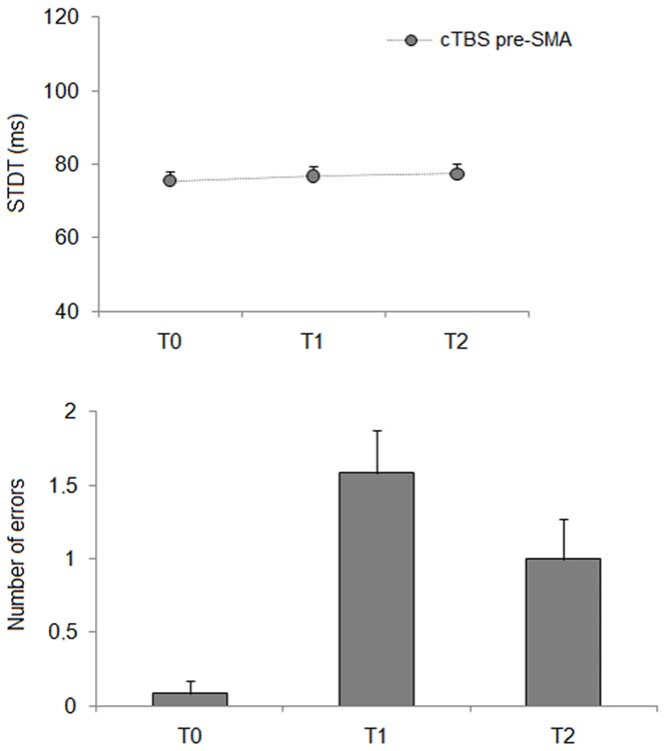
Changes in somatosensory temporal discrimination thresholds (STDT) induced by continuous theta-burst stimulation (cTBS) over pre-supplementary motor area (pre-SMA) in healthy subjects. Each point represents the mean; bars represent standard error. Upper panel: X axis: time: T0 (before cTBS), T1 (5 minutes after cTBS) and T2 (15 minutes after cTBS). Y axis: STDT expressed in millisecond**s**. Lower panel: cTBS-induced changes in the number of errors subjects made during the experimental procedures.

**Effects of cTBS over pre-SMA on the STDT:** Repeated measures ANOVA for STDT showed a non significant effect of factor “time” (F_(2,22)_ = 1.38; *P* = 0.27) (Figure 2). cTBS over pre-SMA therefore left STDT values unchanged. Conversely, Friedman's ANOVA showed that the number of errors during the STDT task changed significantly after cTBS over the pre-SMA (χ^2^ = 13.38; *P* = 0.001). Wilcoxon's test used for post hoc analysis showed that number of errors subjects made during STDT testing increased significantly after cTBS over the pre-SMA at T1 (*P* = 0.004) and T2 (*P* = 0.02) (Figure 2).

**Effects of cTBS over the right DLPFC on the STDT:** Repeated measures ANOVA for STDT showed a non significant effect of factor “time” (F_(2,22)_ = 1.23; *P* = 0.31). Friedman's repeated-measures ANOVA showed no significant differences in the number of errors during the STDT task before and after cTBS.

**Figure 3:**
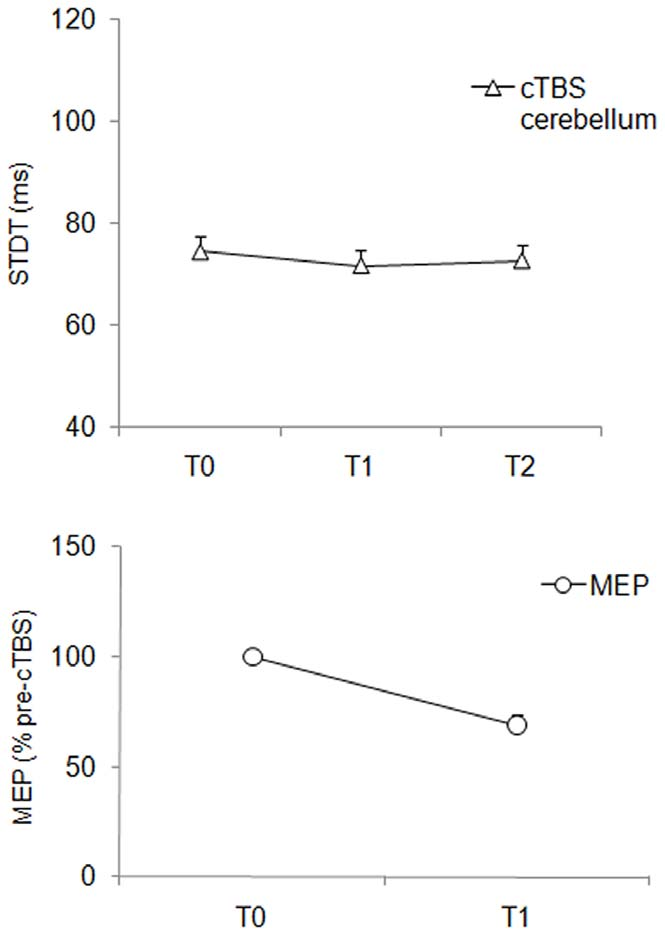
Changes in somatosensory temporal discrimination thresholds (STDT) induced by continuous theta-burst stimulation (cTBS) over left lateral cerebellum in healthy subjects. Upper panel: X axis: time: T0 (before cTBS), T1 (5 minutes after cTBS) and T2 (15 minutes after cTBS). Y axis: STDT expressed in millisecond**s**. Each point represents the mean; bars represent standard error. Lower panel: cTBS-induced changes in motor evoked potential (MEP) size evoked in the right primary motor area (M1). X axis time T0 (before cTBS), T1 (10 minutes after cTBS). Y axis: MEP amplitude expressed as percentage of the MEP at T0.

**Effects of cTBS over the left lateral cerebellum on the STDT:** Repeated measures ANOVA for STDT showed a non significant effect of factor “time” (F_(2,28)_ = 1.15; *P* = 0.32) (Figure 3). Friedman's repeated-measures ANOVA for number of errors during the STDT task showed no significant differences in the number of errors before and after cTBS.

**Figure 4:**
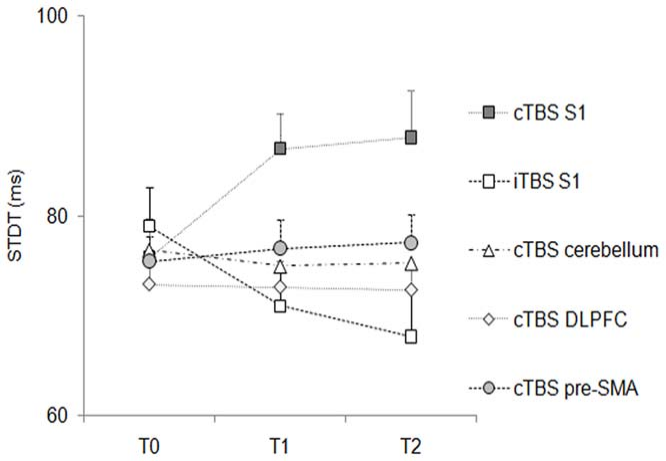
Within-subject changes in somatosensory temporal discrimination thresholds (STDT) induced by continuous theta-burst stimulation (cTBS) over primary somatosensory cortex (S1), pre-supplementary motor area (pre-SMA), left lateral cerebellum, and dorsolateral prefrontal cortex (DLPFC) and intermittent theta-burst stimulation over S1 in healthy subjects. Each point represents the mean; bars represent standard error. X axis: time T0 (before cTBS/iTBS), T1 (5 minutes after cTBS/iTBS), T2 (15 minutes after cTBS/iTBS); Y axis: STDT expressed in milliseconds.

**Within-subjects changes in STDT values across different cortical areas:** In the 12 subjects who underwent all the experimental sessions, repeated measures ANOVA showed a significant effect of factor “cortical areas” (F_(4,44)_ = 2.99; *P* = 0.02) and a significant interaction of factors “cortical areas” and “time” (F_(2.7,29.8)_ = 8.45; *P* = 0.0004 corrected for non sphericity) (Figure 4). Repeated measures ANOVA comparing STDT values at T0 collected in the 12 subjects in each experimental session showed that STDT values at T0 remained statistically unchanged across the experimental sessions (F_(4,44)_ = 0.79; *P* = 0.53).

### Control experiments

**Figure 5:**
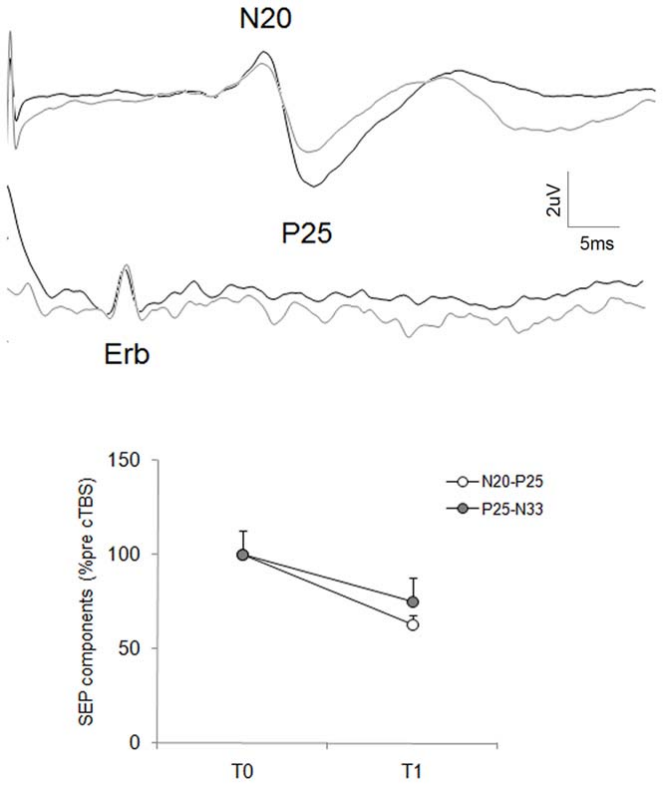
Changes in upper limb somatosensory evoked potentials (SEP) after continuous theta-burst stimulation (cTBS) over primary somatosensory cortex (S1) in healthy subjects. Upper panel: traces of upper limb SEP in a representative healthy subject before (black line) and 10 minutes after cTBS over S1 (gray line). Each trace represents the average of 500 responses. Lower panel: Changes in SEP components before and after cTBS over S1 in healthy subjects. Each point represents the mean and the bars represent standard error. X axis time T0 (before cTBS), T1 (10 minutes after cTBS). Y axis: SEP amplitude expressed as percentage of the SEP at T0.

**Effects of cTBS over right S1 on the upper-limb SEP:** In the 10 subjects who underwent SEP recordings before and after cTBS over S1, ANOVA for the amplitude of N20-P25 and P25-N33 after cTBS showed that both SEP components decreased significantly in size after cTBS [factor “time” (N20-P25: F_(1,9)_ = 44.2; *P*<0.0001; P25-N33: F_(1,9)_ = 7.84; *P*<0.02)] (Figure 5). Conversely, N20 amplitude remained statistically unchanged after cTBS over S1 (F_(1,9)_ = 3.63; *P* = 0.09).

**Effects of cTBS over left lateral cerebellum on contralateral M1 excitability:** In the 10 subjects who underwent contralateral M1 excitability testing, ANOVA for MEP size showed a significant decrease in the MEP size evoked on the right M1 [factor “time” (F_(1,9)_ = 18.35; *P* = 0.002)] (Figure 3, lower panel).

## Discussion

In this study in healthy subjects, TBS provided the information we sought on the specific cortical areas intervening in the STDT. cTBS and iTBS applied over the right S1 induced opposite changes in the STDT: after cTBS, STDT values increased (worsened) whereas after iTBS they decreased (improved). Unexpectedly, cTBS over the pre-SMA left the STDT statistically unchanged but it increased the number of errors subjects made in distinguishing trials testing a single stimulus and those testing paired stimuli. cTBS over the left lateral cerebellum and right DLPFC left the STDT unchanged. The cortical and subcortical areas thought to intervene in other forms of temporal processing therefore only partially overlap with those responsible for the STDT.

Our experimental procedures envisaged several precautions to avoid methodological errors. The similar baseline STDT values in each experimental session not only exclude a possible learning bias but also confirm that the psychophysical variable we studied, the STDT, yields reproducible data. Further evidence excluding attentional-related changes came from the “catch trials” showing that the number of errors remained statistically unchanged in the “S1” session. Because we used a neuronavigation system the lack of changes in STDT after cTBS over pre-SMA ad DLPFC presumably did not depend on an erroneous coil localization over the scalp. The decreased SEP parietal components after cTBS over S1 and the decreased MEP after cTBS over left lateral cerebellum also provide further evidence that our stimulating protocol effectively inhibited neuronal activity in the right S1 and in the left cerebellar hemisphere.

Our finding that cTBS over the right S1 increases whereas iTBS over the same cortical area decreases STDT values provides new evidence suggesting that the right S1 plays a prominent encoding role in the STDT. Our STDT findings agree with those reported in studies by Bolognini et al. [7] and Hannula et al. [8] showing that single-pulse TMS interferes online with STDT processing and therefore suggest that the cortical area encoding STDT is S1. In our study, further underlining the importance of S1 in STD processing we provide evidence that plasticity-inducing protocols can alter (improve or worsen) STDT values. Because TBS induces changes in LTP/LTD-like synaptic plasticity [9], [19], [38], [39] and modulates the STDT for at least 20 minutes after TBS ends, we suggest that changes in the STDT depend on changes in S1 synaptic activity. Some help in interpreting our results comes from Tamura et al.'s observation [5] that in patients with focal hand dystonia the altered STDT correlates with altered somatosensory intracortical inhibition. Although our study provides no direct data about the synaptic mechanisms involved in STDT encoding, animal experiments show that fast-spiking inhibitory interneurons, engaged monosynaptically by thalamocortical inputs, exert powerful feed forward-inhibition on the post-synaptic cortical neuron cell bodies in S1 [40], [41]. The STDT might therefore result from the gating and coordinating functions of the fast-spiking inhibitory interneurons recruited by the thalamo-cortical input. cTBS and iTBS over S1 could promote homeostatic changes in synaptic activity that in turn modulate activity in the inhibitory interneurons. These findings find some support from the State-dependent Network Model (SDN) [42], [43]. This model, in contrast with those postulating a single centralized internal clock [44], proposes that timing is an ubiquitous neural computational component, and because neural networks are naturally complex structures endowed with time-dependent properties they can inherently process temporal inputs. We therefore hypothesize that S1 is specifically involved in early somatosensory stimuli timing and cTBS probably increases the STDT in healthy subjects by depressing activity in S1 cortical neural circuits. Because cTBS over S1 significantly decreased N20-P25 and P25-N33 amplitudes but left N20 amplitude unchanged we suggest that cTBS-induced changes in N20-P25 depend mainly on changes in P25. SEP N20 component is generated at some depth from the cortical surface (BA 3b) whereas the P25 and N33 components involve generators in superficial area 1 [45]–[47]. Consistent with a previous study [38], our findings therefore suggest that cTBS over S1 modulates neuronal activities within superficial areas (BA 1) of S1. Our finding that iTBS over S1 improved STDT values in healthy subjects substantiates the putative role of S1 cortex in STD processing. Because iTBS over S1 left the number of errors unchanged we exclude the possibility that iTBS improved our participants' attention levels and in turn STDT values. In line with our finding that iTBS decreases the STDT, others showed that 5 Hz rTMS [48], [49] or iTBS [19] over S1 enhances tactile spatial discrimination – another form of sensory discrimination whose physiological mechanisms differ from those involved in the STDT.

Our findings seemingly contrast with those from Pastor et al. [6] who reported selective pre-SMA cortical activation during STD tasks. In the experimental procedures during their fMRI study, however, subjects had to press a button as soon as they perceived paired stimuli. Because the pre-SMA is also involved in motor preparation and execution, the pre-SMA activation they found during the STD procedures might conceivably at least in part reflect task-related motor activity. This theoretical explanation notwithstanding, evidence implying that the pre-SMA cortex contributes to the STDT receives support from the increased number of errors our subjects made in the discriminative task after cTBS. Hence rather than playing an encoding role, the pre-SMA could help integrate the interplay between the cortical and subcortical structures. An alternative explanation is that TBS could modulate neural pathways from the pre-SMA to prefrontal cortex, thus impairing the subjects' attentiveness. This hypothesis is however unlikely insofar as cTBS over the DLPFC changed neither the STDT nor the number of errors subjects made during the discriminative task.

Using a different experimental approach studies using TMS and time reproduction tasks to investigate sensory system time processing suggested that dorsolateral prefrontal cortex (DLPFC) [11] and cerebellum [35], [50] play a role in temporal processing. Given the DLPFC's reported role in temporal processing [51], when we investigated how the DLPFC contributed to STDT, we found, as expected, that cTBS applied on the right DLPFC induced no detectable changes in the STDT. Previous studies using fMRI reported DLPFC activation (BA 9, 10, 46) during tasks such as item recognition, free recall for verbal items, spatial and object storage [52] and sequential-letter memory tasks [53]. Another factor that could change the way the DLPFC contributes to temporal analysis is how long the tested ISIs last. For example, Rammsayer and Lima [54] found that a secondary cognitive task leaves temporal processing for ISIs ranging from 50 to 100 msec unaffected but impairs temporal processing for longer ISIs (in the range of seconds). Also, others testing 4 second ISIs found that patients with lesions involving the DLPFC showed a significant timing deficit [55]. Collectively, these data suggest that the DLPFC, a brain region known to be important for working memory, could intervene in cognitive controlled time measurement but may be unessential for temporal processing involving short ISIs (tens of milliseconds), a task requiring highly perceptual discrimination not accessible to cognitive control [11]. Our observation that in the same subject cTBS/iTBS over S1 but not cTBS over DLPFC modulates the STDT, supports the hypothesis that the cortical networks engaged in time reproduction tasks – entailing memory formation processes – differ from those involved in the STDT. This difference underlines the need to define the precise type of altered temporal processing of sensory information in patients with neurological diseases.

Because the cTBS-induced inhibition in the left lateral cerebellum modulated activity in the contralateral M1 but had no effect on STDT values, we suggest that the cerebellum probably plays no detectable role in temporal processing as tested with the temporal discrimination task we used. Investigating purely perceptive tasks (subjects had to compare the interval duration between two stimuli), Ivry et al. [12] and Harrington et al. [56] found that cerebellar lesions were associated with altered time perception tasks. rTMS studies [35], [50], [57] also demonstrated that the lateral cerebellum is implicated in temporal processing. Experiments designed to estimate the interval duration or compare the duration of two ISIs use procedures that activate neural structures other than those underlying the STDT, or investigate different intervals (seconds-hundreds of milliseconds vs tens of milliseconds).

Our within-subjects study in healthy subjects shows that the STDT, unlike other temporal discriminative tasks involving working memory processes, is specifically encoded in S1, possibly depends on intrinsic properties in cortical neural circuits and can be modulated by TBS protocols. We also conclude that other cortical (pre-SMA, DLPFC) and subcortical areas (left lateral cerebellum) play a less prominent role in the STDT than S1. This new information giving further insight into the mechanisms involved in temporal discrimination of tactile stimuli – as tested with the STDT- in healthy subjects – helps interpret the sensory processing deficits in neurological diseases such as focal dystonia and Parkinson's disease and possibly prompts future studies applying TBS over S1 for therapeutic purposes in dystonic patients [15]–[18].
